# Serines 440 and 467 in the Werner syndrome protein are phosphorylated by DNA-PK and affects its dynamics in response to DNA double strand breaks

**DOI:** 10.18632/aging.100629

**Published:** 2014-01-14

**Authors:** Rika Kusumoto-Matsuo, Deblina Ghosh, Parimal Karmakar, Alfred May, Dale Ramsden, Vilhelm A. Bohr

**Affiliations:** ^1^ Laboratory of Molecular Gerontology, National Institute on Aging, NIH, Baltimore, MD 21224, USA; ^2^ Department of Life Science and Biotechnology, Jadavpur University, Kolkata 700 032, India; ^3^ Department of Biochemistry and Biophysics, Lineberger Comprehensive Cancer Center, and Curriculum in Genetics and Molecular Biology, and University of North Carolina at Chapel Hill, NC 27599, USA

**Keywords:** Werner, WRN, DNA-PK, serine phosphorylation, DSB, etoposide

## Abstract

WRN protein, defective in Werner syndrome (WS), a human segmental progeria, is a target of serine/threonine kinases involved in sensing DNA damage. DNA-PK phosphorylates WRN in response to DNA double strand breaks (DSBs). However, the main phosphorylation sites and functional importance of the phosphorylation of WRN has remained unclear. Here, we identify Ser-440 and −467 in WRN as major phosphorylation sites mediated by DNA-PK. *In vitro*, DNA-PK fails to phosphorylate a GST-WRN fragment with S440A and/or S467A substitution. In addition, full length WRN with the mutation expressed in 293T cells was not phosphorylated in response to DSBs produced by bleomycin. Accumulation of the mutant WRN at the site of laser-induced DSBs occurred with the same kinetics as wild type WRN in live HeLa cells. While the wild type WRN relocalized to the nucleoli after 24 hours recovery from etoposide-induced DSBs, the mutant WRN remained mostly in the nucleoplasm. Consistent with this, WS cells expressing the mutants exhibited less DNA repair efficiency and more sensitivity to etoposide, compared to those expressing wild type. Our findings indicate that phosphorylation of Ser-440 and −467 in WRN are important for relocalization of WRN to nucleoli, and that it is required for efficient DSB repair.

## INTRODUCTION

Werner Syndrome (WS) is an autosomal recessive disorder characterized by premature aging, elevated genomic instability and increased cancer [1]. Cells from WS patients have a reduced replicative life span [2]. WS cells exhibit genomic instability with DNA deletions, insertions, and rearrangements [3]. In addition, WS cells show hypersensitivity to some DNA damaging agents, including 4-nitroquinoline-1-oxide (4NQO) and topoisomerase inhibitors as well as mild sensitivity to ionizing radiation [4, 5]. The WRN protein mutated in WS possesses an exonuclease domain, a multimerization region, an acidic region, a helicase domain, a RecQ conserved (RQC) domain, a helicase and ribonuclease D/C-terminal (HRDC) domain and a nuclear localization signal (NLS) [6, 7].

WRN localizes to nucleoli under normal conditions, and translocates to the nucleoplasm in response to stress. It is proposed that post-translational modifications of WRN such as phosphorylation, acetylation, and sumoylation occur in response to stresses and affect its dynamics [8]. WRN is phosphorylated at serine, threonine or tyrosine residues by several kinases in response to DNA damage or stress. Tyrosine kinase, c-Abl-mediated phosphorylation of WRN after bleomycin treatment or after replication stress has been correlated to WRN delocalization from nucleoli to DNA damage-induced foci in the nucleoplasm [9]. WRN is also phosphorylated in an ATR/ATM dependent manner in response to DNA replication arrest or DNA damage during S phase of the cell cycle [10]. Suppression of ATR-mediated phosphorylation of WRN prevents proper accumulation of WRN in nuclear foci, causing breakage of stalled forks [10]. Inhibition of ATM-mediated phosphorylation of WRN leads to retention of WRN nuclear foci, resulting in reduced viability after fork collapse[10]. Thus, phosphorylation of WRN by c-Abl, ATR or ATM affects translocation of WRN.

DNA-dependent kinase (DNA-PK) also targets WRN *in vivo* after treatment with regents that cause DNA double strand breaks (DSBs) [5, 11]. DSBs are cytotoxic lesions that can lead to mutation or cell death if not repaired proficiently. In mammalian cells, there are two major pathways for DSB repair: non-homologous end joining (NHEJ) and homologous recombination (HR)[12]. The DNA-PK complex, comprised of the DNA-PK catalytic subunit (DNA-PKcs) and the Ku 70/86 heterodimer, is an essential factor for NHEJ in mammalian cells and telomere maintenance, together with the XRCC4/DNA ligase IV (X4L4) complex [13-18]. Previous studies indicate that WRN interacts with NHEJ factors, and that its enzymatic activities are affected by the interaction. Ku 70/86 is one of the most prominent protein-interactors of WRN, and it promotes WRN exonuclease activity [19, 20]. The X4L4 complex binds to WRN and alters its exonuclease activity [21]. WRN also accumulates at laser-induced DSBs [22]. Together, these data suggest a role for WRN phosphorylation in the repair of DSBs. Ser-319 was identified as a singular and unique phosphorylation site by DNA-PK within WRN (1-333) [7]. The serine is located proximal to a WRN multimerization region, and the phosphorylation at this site affects neither exonuclease activity nor multimeric state [7]. Phosphorylation residues for DNA-PK in other regions of WRN in response to DSBs have not yet been identified.

In this study, we asked whether WRN is phosphorylated by DNA-PK at other residues in response to DSBs, and whether the phosphorylation affects its translocation in cells. In comparison with wild type WRN, we analyzed the localization of phosphorylation mutants of WRN in response to DSBs produced by micro irradiation in the nucleus of human living cells. We also analyzed the sensitivity of WS cells overexpressing WRN phosphorylation mutants to DSBs produced by etoposide.

## RESULTS

### DNA-PK phosphorylates WRN within the putative acidic repeats and in the C-terminus

**Figure 1:**
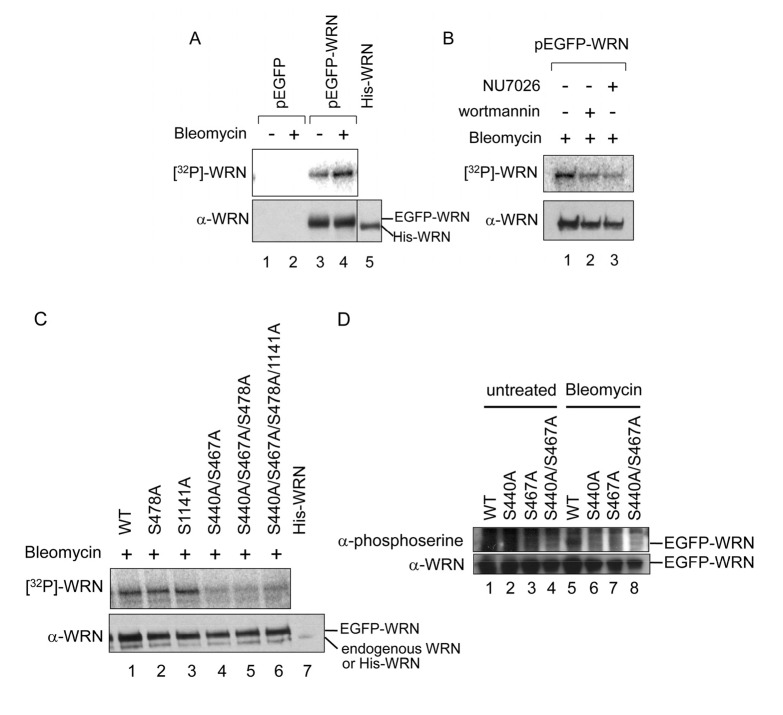
Mapping DNA-PK phosphorylation sites in WRN (**A**) Schematic representation of His- or GST-tagged WRN fragments used in *in vitro* phosphorylation assay. (**B** and **C**) *In vitro* phosphorylation assay. Purified His- or GST-tagged WRN fragments were incubated with purified DNA-PKcs, Ku 70/86, and activated DNA in the presence of [γ-32P]ATP. Amido black staining is shown (**B**). The phosphorylation was visualized (**C**). *Asterisk* indicates the GST (500-946) fragment. Note that GST (239-499) migrated slower because of many acidic amino acids.

To map the region of WRN that is phosphorylated by DNA-PK, we first performed *in vitro* phosphorylation assays using a series of WRN fragments (Fig. 1). The WRN fragments are shown in Fig. 1A. These fragments were partially purified from *E. coli* using His- or GST-tags, and incubated with purified DNA-PKcs and Ku 70/80 in the presence of activated DNA and [γ-^32^P]ATP. The samples were subjected to SDS-PAGE and amido black staining, and the phosphorylation was visualized (Figs. 1B and 1C). GST itself was not phosphorylated by DNA-PK (Fig. 1C, lane 6). We found that the phosphorylation sites were located in the acidic region of WRN (239-499), and in the C-terminal domain of WRN (949-1432) (Fig. 1C, lanes 3 and 5). The signal from WRN (239-499) was much stronger than that of WRN (949-1432), suggesting that a major phosphorylation site or multiple phosphorylation sites are located in the acidic region. For fine mapping of WRN phosphorylation sites in the C-terminal domain, a truncated WRN (949-1236) was examined further, and since it was not phosphorylated, the minor phosphorylation site(s) were likely located in WRN (1237-1432) (supplementary Fig. S1).

We also analyzed phosphorylated WRN by mass spectrometry and identified the amino acids. Recombinant full length WRN purified from Sf9 cells was phosphorylated *in vitro* by DNA-PK, and subjected to SDS-PAGE. Full length WRN was excised from the gel and subjected to in-gel trypsin digestion. The trypsinized samples were enriched for phospho-peptides using an immobilized metal affinity column (IMAC) and the enriched peptide mixtures were analyzed using LC-MS/MS. We obtained four peptides, STEHLSPNDNENDTSYVIESDEDCEME (421-447), HLSPNDNENDTSYVIESDEDLEMEMLK (424-450 and/or 451-477), SLENLNSGTVEPTHSK (478-493) and AYSSSQPVISAQEQETQIVLYGK (1137-1159), containing serine as a phosphorylated candidate (underlined). Note that the HLSPNDNENDTSYVIESD LEMEMLK peptide may originate from 424-450 and/or 451-477, because 424-477 consists of two tandem repeats of 27 amino acids. The results suggested that Ser-440, −467, −478 or −1141 might be phosphorylated in the *in vitro* phosphorylation assay. Ser-440 and −467 are located in the acidic repeat, and Ser-478 is located just after the repeats (supplementary Fig. S2). This is consistent with the results from the *in vitro* phosphorylation assay (Fig. 1C). Ser-1141 is also a candidate for phosphorylation based on the result of the LC-MS/MS analysis. However, WRN (949-1236) was not phosphorylated *in vitro* (supplementary Fig. S1).

### Ser-440 and −467 are phosphorylated in vivo by DNA-PK in response to bleomycin treatment

**Figure 2:**
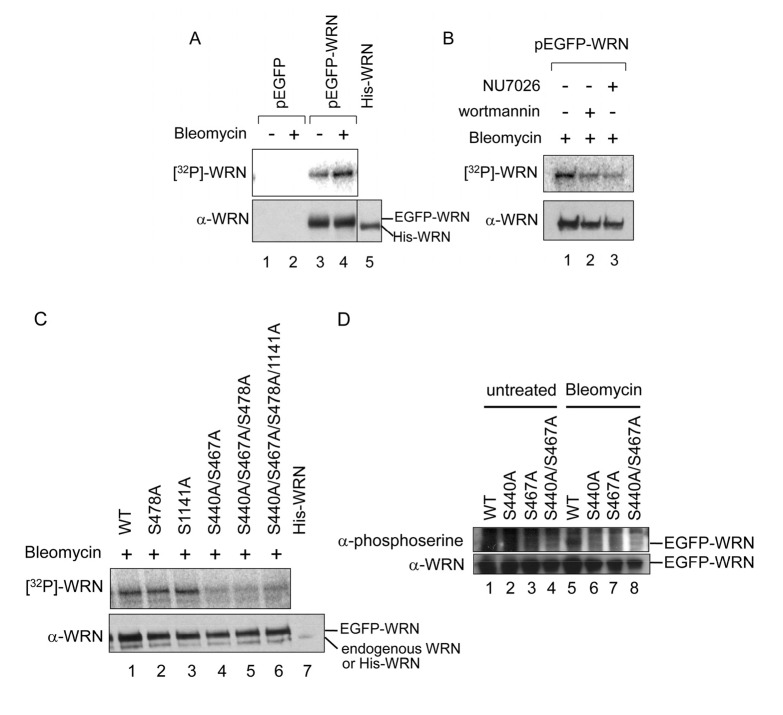
Bleomycin induces WRN phosphorylation at Ser-440 and 467 by DNA-PK (**A**-**D**) *In vivo* phosphorylation assay with radio-labeling. (**A**) Empty vector (pEGFP) (lanes 1 and 2) and pEGFP-WRN (lanes 3 and 4) were transfected to 293T cells. The cells were incubated in the absence (lanes 1 and 3) or presence (lanes 2 and 4) of 5 μg/ml bleomycin and [^32^P] labeled phosphate. WRN proteins were immunoprecipitated. Recombinant His-tagged full length WRN (800 ng) was loaded to assign the position of endogenous WRN (lane 5). The phosphorylated proteins were visualized (upper panel), followed by Western blotting with anti-WRN antibody (lower panel). (**B**) 293T cells transfected with pEGFP-WRN were treated with 5 μg/ml bleomycin in the presence of PI-3 kinase inhibitors, 25 μM wortmannin (lane 2) and 20 μM NU7026 (lane 3). (**C**) 293T cells transfected with pEGFP-WRN [wild type (WT) or mutant as indicated] were treated with or without 5 μg/ml bleomycin as indicated. Recombinant His-tagged full length WRN was loaded (lane 7). (**D**) *In vivo* phosphorylation assays using anti-phosphoserine antibody. HEK293 cells transfected with pEGFP-WRN (WT or mutant as indicated) were treated with (lanes 5-8) or without 5 μg/ml bleomycin (lanes 1-4),. EGFP proteins were immunoprecipitated with anti-GFP antibody. The phosphorylated proteins were detected by Western blotting with anti-phosphoserine antibody. The membrane was deprobed and analyzed by Western blotting with anti-WRN antibody.

To address whether phosphorylation at Ser-440, −467, −478 or −1141 takes place *in vivo*, we performed an *in vivo* phosphorylation assay. 293T cells were transfected with a vector to overexpress N-terminally EGFP-tagged WRN and incubated in the presence of [^32^P] labeled orthophosphoric acid and bleomycin to introduce DSBs. Cells were then lysed and WRN was immuno-precipitated. The products were subjected to SDS-PAGE and transferred to a PVDF membrane. First, we tested whether exogenous WRN was phosphorylated *in vivo* in response to DSBs. To distinguish EGFP-WRN from endogenous WRN, we loaded recombinant full length His-tagged WRN, which migrated at almost the same position as endogenous WRN. However, endogenous WRN was not detected in this experiment (Fig. 2A, lanes 1, 2 and 5). EGFP-WRN was detected and was phosphorylated more in the presence of bleomycin than in its absence, suggesting that exogenous WRN is phosphorylated in response to bleomycin (Fig. 2A, compare lanes 3 and 4). Next, we tested whether bleomycin induced-phosphorylation was DNA-PK dependent. Cells overexpressing EGFP-WRN were treated with bleomycin in the presence of either the PI-3 kinase inhibitor wortmannin (IC_50_=5 μM for DNA-PK and ATM, IC_50_>100 μM for ATR) or the DNA-PK inhibitor NU7026 (Fig. 2B). In the presence of either of these inhibitors, EGFP-WRN was less phosphorylated *in vivo*, suggesting that the phosphorylation of exogenous WRN was at least partially DNA-PK-dependent (Figs. 2B). To determine which serine among 440 or 467, 478 and 1141 was phosphorylated by DNA-PK *in vivo*, we introduced Ala mutations to the pEGFP-WRN vector at those sites individually or in combination, and then performed *in vivo* phosphorylation assays (Figs. 2C and 2D). EGFP-WRN S1141A was phosphorylated at almost the same level as the EGFP-WRN wild type, and so was EGFP-WRN S478A, suggesting that Ser-478 and −1141 were not phosphorylated *in vivo*. EGFP-WRN S440A/S467A, EGFP-WRN S440/S467A/S478A, and EGFP-WRN S440/S467A/S478A/S1141A were barely phosphorylated, suggesting that either one or both Ser- 440 and −467 were responsible for the phosphorylation. We detected a small amount of endogenous WRN or degraded EGFP-WRN, distinct from EGFP-WRN (Figs. 2C). Next, we introduced a mutation at either of these two serines. HEK293 cells were transfected with these vectors, and incubated in the presence of bleomycin to introduce DSBs. Cells were then lysed and GFP-WRN was immunoprecipitated. The products were subjected to SDS-PAGE and transferred to a PVDF membrane. Phosphorylated WRN was detected using anti-phosphoserine antibody (Fig. 2D). EGFP-WRN wild type, but not S440A, S467A and S440A/467A was phosphorylated in response to bleomycin (Fig. 2D). Based on these experiments, we concluded that WRN is phosphorylated at both Ser-440 and −467 by DNA-PK in response to bleomycin treatment *in vivo*.

### Ser-440 and −467 are phosphorylated in vitro by DNA-PK

**Figure 3:**
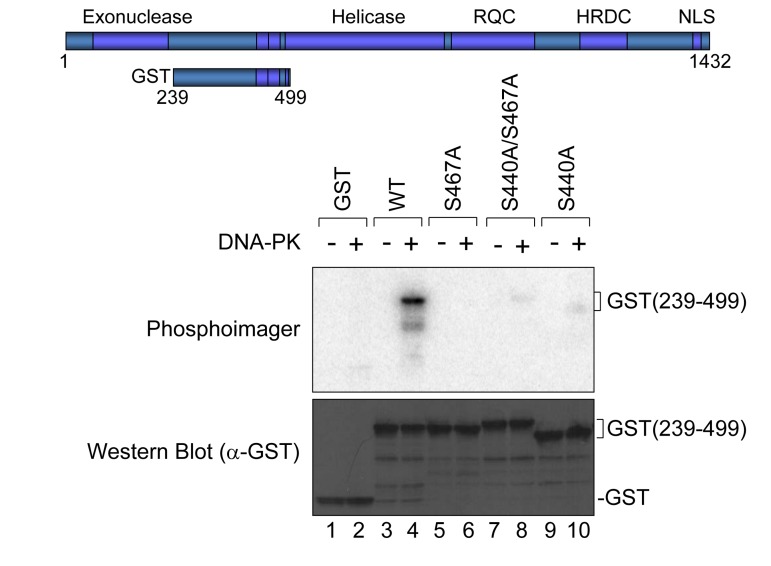
*In vitro* phosphorylation at Ser-440 and −467 by DNA-PK GST-tagged WRN fragment (239-499) as schematically represented was used. Purified GST or GST-tagged fragment with or without Ala substitution at Ser-440 and/or −467 was incubated with purified DNA-PKcs, Ku 70/86, and activated DNA in the presence of [γ-^32^P]ATP. Phosphorylation was visualized (upper panel). An immunoblot with anti-GST antibody is shown (lower panel).

Experiments were then performed to confirm that Ser-440 and −467 are phosphorylated *in vitro*. The GST-WRN (239-499) fragment with alanine substitution at Ser-440 and/or −467 was expressed in bacterial cells and partially purified. The mutated WRN fragment was utilized in *in vitro* phosphorylation assays (Fig. 3). The products were subjected to SDS-PAGE and transferred to a PVDF membrane. The fragment without mutation, but not the one with S440A and S467A substitution was phosphorylated by DNA-PK (Fig.3, lanes 4 and 8). Consistent with results in Fig. 2D, neither S440A nor S467A single mutant was phosphorylated (Fig. 3, lanes 6 and 10), suggesting that Ser-440 is required for phosphorylation of Ser-467 and that Ser-467 is required for phosphorylation of Ser-440. We also did not detect phosphorylation of the GST-WRN (239-499) fragment containing Ser-319, which is another residue phosphorylated by DNA-PK. The N-terminal region of WRN may be required for the phosphorylation, since WRN (1-333) was used for the Ser-319 phosphorylation experiments [7]. Taken together with the results from the *in vivo* phosphorylation assay, Ser-440 and −467 are major phosphorylation sites by DNA-PK both in *in vitro* and *in vivo*.

### The WRN phosphorylation at Ser-440 and −467 is not required for its accumulation at DSBs

**Figure 4:**
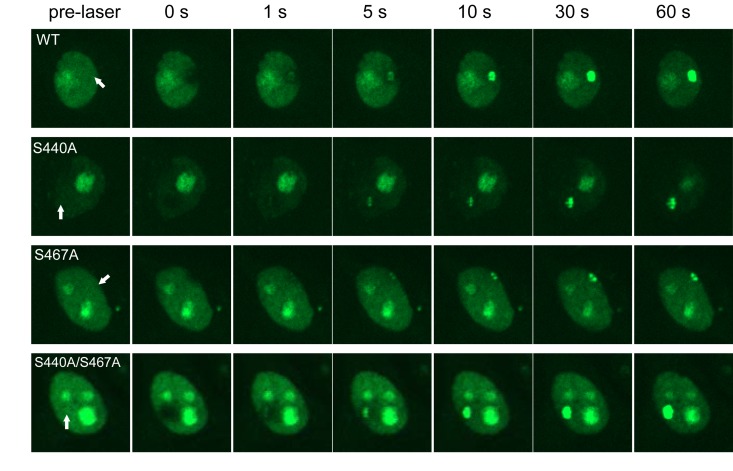
Accumulation of WRN wild type and phosphorylation mutants at laser-induced DSBs HeLa cells overexpressing either EGFP-WRN wild type (WT) or mutant (S440A, S467A or S440A/S467A) were laser-irradiated at the sites indicated by arrows. Time-dependent accumulation of EGFP-WRN WT and mutants at the DSBs sites were shown.

To understand the biological significance of WRN phosphorylation at Ser-440 and −467, we analyzed WRN translocation within the nucleus using laser microirradiation and confocal microscopy. WRN localizes in nucleoli under normal conditions, and leaves the nucleoli to form foci in response to various stresses. Previously Lan *et al.* reported that GFP-WRN accumulated at sites of DSBs and the fluorescence at the sites reached a plateau within 3 min [22]. To set up conditions to produce DSBs by laser irradiation, we irradiated HeLa cells with the laser at several doses, and immunostained for γH2AX. We observed γH2AX foci at the site of irradiation using 14% intensity with a 435 nM laser (data not shown). HeLa cells were transfected with vectors to overexpress EGFP-WRN wild type, S440A, S467A or S440A/S467A mutants, and microirradiated at 14% intensity with the 435 nM laser to induce DSBs. EGFP-WRN wild type localized at nucleoli before irradiation, and accumulated at the sites of irradiation in a time-dependent manner. Within a second after irradiation WRN accumulated at the irradiated sites and fluorescence at the sites reached a plateau 30 seconds after irradiation (Fig. 4, upper panels). All of the phosphorylation mutants, EGFP-WRN S440A, S467 and S440A/S467A, also localized at nucleoli before irradiation, and accumulated at the irradiated site with the same kinetics as wild type. We confirmed that accumulation kinetics of the EGFP- WRN wild type and S467A was similar by quantifying and plotting of the fluorescence signals up to 60 seconds (supplementary Fig. S3). These results indicated that phosphorylation of WRN by DNA-PK is not required for its accumulation at the site of DSBs produced by laser irradiation.

### Ser-440 and −467 are required for WRN relocalization to nucleoli

**Figure 5:**
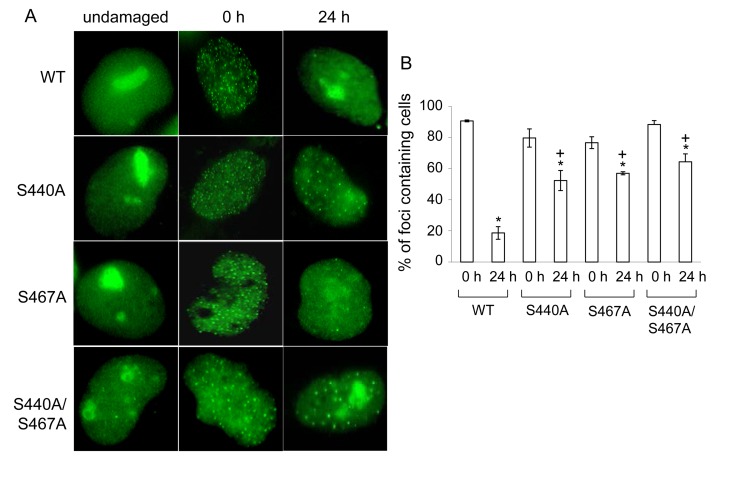
WRN but not phosphorylation mutant relocalizes to the nucleoli post etoposide exposure (**A**) AG11395 cells overexpressing either EGFP-WRN wild type (WT) or mutant (S440A, S467A or S440A/S467A) were incubated with 35 μM etposide for 3 hours. Cells were fixed and EGFP signals were visualized before and after incubation for another 24 hours in fresh medium. Representive images are shown. (**B**) The percent of cells containing WRN foci. At least 100 cells were scored at each time point. The average of three independent experiments with standard deviation is plotted. Asterisks (*) indicate significant difference between 0 h and 24 h (p<0.05). Plus (+) indicate significant difference between Wild type and mutants.

After the intensity of fluorescence of EGFP-WRN reached a plateau in the irradiated sites, it persisted at least for 4 hours after the laser irradiation[22]. Consistent with this, EGFP-WRN wild type and mutants persisted at the site of DSBs up to 1 hour in HeLa cells (data not shown). To address whether phosphorylation of WRN by DNA-PK affects its retention at the foci, we analyzed the accumulation of exogenous EGFP-WRN or the phosphorylation mutants in WS cells after 24 hours-recovery from exposure to etoposide, a DNA topoisomerase II inhibitor, which produces DSBs irrespective of DNA replication (Fig. 5A). All of the phosphorylation mutants as well as the wild type localized to nucleoli without etoposide exposure (Fig. 5A, undamaged). Consistent with a previous report [23], the wild type, as well as S440A, S467A, and S440A/S467Amutants formed foci in the nucleoplasm upon exposure to etoposide (Fig. 5A, 0 h). After the recovery, wild type WRN relocated to nucleoli, whereas many more foci of the phosphorylation mutants of WRN remained in the nucleoplasm (Fig. 5A, 24 h). The percentage of cells showing EGFP-WRN wild type foci in the nucleoplasm decreased to 20% after 24 hours-recovery from etoposide-induced damage, whereas for S440A 66% cells, for S467A 75% cells and for S440A/S467A 73% cells showed foci at the nucleoplasm after the recovery (Fig. 5B). These data suggested that the major phosphorylation sites, Ser-440 and −467, are required for the efficient relocalization of WRN to nucleoli.

### Phosphorylation of Ser-440 and −467 leads to decreased cellular sensitivity to etoposide

**Figure 6:**
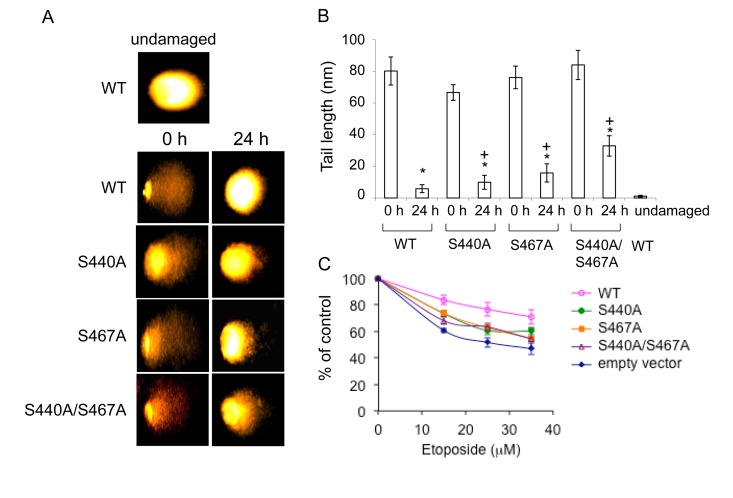
Cells overexpressing the phosphorylation mutants are moderately sensitive to etoposide (A) Comet assay with AG11395 cells overexpressing either EGFP-WRN wild type (WT) or mutant (S440A, S467A or S440A/S467A) 24 h after 35 μM etoposide treatment. Representive images are shown. (B) Tail length in (A) are indicated. At least 17 cells were measured. Asterisks (*) indicate significant difference between 0 h and 24 h (p<0.05). Plus (+) indicate significant difference between Wild type and mutants. (C) AG11395 cells overexpressing either EGFP-WRN wild type (WT), S440A, S467A or S440A/S467A were treated with 0, 15, 25 or 35 μM etoposide. 24 h after the treatment, cell proliferation was evaluated by the MTT assay. Significant difference between cells expressing empty vector and WT, or three mutants at concentration of 35 μM (p<0.05).

To test whether retained WRN phosphorylation mutant foci represent non-repaired DSBs, we performed alkaline comet assays. WS cells expressing EGFP-WRN wild type or mutants were treated with 35 μM etoposide for 3 hours, were allowed to recover for 24 hours, and were assayed. As expected, DSBs were decreased in cells expressing either type of WRN after recovery for 24 hours (Fig. 6A and B, compare 0 h and 24 h). However, there was an increase in residual DSBs in cells overexpressing either of the WRN mutants after 24 hour-recovery. We also examined cytotoxicity by etoposide by evaluating cell proliferation by the MTT assay (Fig. 6C). Cells were treated with 15-35 μM etoposide for 3 hours, and were allowed to recover for 24 hours. WRN wild type overexpressing WS cells exhibited a significant decrease in sensitivity to etoposide toxicity, compared to WS cells transfected with empty vector. WS cells expressing either of phosphorylation mutants exhibited a slight decrease in sensitivity, compared to the one transfected with empty vector. These results suggested that phosphorylation of WRN contributes to the protection of cells from DSBs produced by etoposide.

## DISCUSSION

We have identified Ser-440 and −467 of WRN as DNA-PK phosphorylation sites both *in vitro* and *in vivo* using bleomycin to produce DSBs. WRN has putative acidic repeats in 424-477, and Ser-440 and −467 are located in the first and second acidic repeat, respectively. We overexpressed WRN mutants with substitution of Ser-440 and/or −467 to Ala, examined the response to laser-induced DSBs in live HeLa cells, and found similar accumulation of all mutants to at DSBs as wild type. Also, the accumulated mutants as well as wild type WRN resided up to an hour after irradiation (data not shown). This is consistent with a report suggesting that the HRDC (helicase and RNaseD C-terminal) domain (1021-1432) was necessary and sufficient for the accumulation to DSBs [22]. We examined the WRN localization after 24 hour-recovery from etoposide-induced DNA damage in WS cells. Interestingly, WRN wild type translocated back to nucleoli, whereas the phosphorylation mutants persisted as nucleoplasmic foci, suggesting that Ser-440 and −467 contribute to the relocalization of WRN to nucleoli. We found that more DSBs persisted in WS cells expressing WRN mutants compared to wild type after 24 hour-recovery from etoposide exposure. Inhibition of proliferation of these cells by etoposide was also tested by the MTT assay. In our experiments, the WS cells expressing EGFP-WRN showed decreased sensitivity to etoposide, compared to those transfected with empty vector (Fig. 6), consistent with a previous report[24], though inconsistent results have been reported about sensitivity of WS cells to etoposide. In another study, they utilized WRN knockdown osteosarcoma cells and 50 % of cells died after exposure to 10 μM of etoposide[25]. In our case, we utilized WRN deficient fibroblast, where no wild type WRN protein exists, and much higher concentrations of etoposide were used (up to 40 μM). A small amount of WRN may be enough to repair etoposide-induced DSBs. Additionally DSBs produced by such higher concentration of etoposide are repairable as we observed in our comet assay (Fig.6). Based on current and previous data, we hypothesize a model for WRN at DSBs. Once DSBs are formed, the HRDC domain of WRN may either bind to the DSBs or interact with other proteins present at DSBs. WRN recruitment is not dependent on Ku, which binds to DSBs at an early step during NHEJ and has strong affinity to WRN[22]. When both DNA-PK and WRN are at the sites of DSBs, WRN Ser-440 and/or −467 are phosphorylated by the kinase. The phosphorylation of WRN may regulate its catalytic activities [11], and may also help recruit other proteins for proficient repair. Thus, fine tuning between DSB repair and regulation of catalytic activities of WRN is essential for DNA repair explaining the genomic instability observed in WS patients. How this regulation contributes to in vivo DNA repair remains speculative but we have clearly found specific target sites of DNA-PK on WRN. It seems likely from our observations that restoration of WRN to nucleoli indicates completion of DNA repair. It is evident from many previous studies that WRN relocation from nucleoli to the nucleoplasm represents an important aspect of the cellular role of WRN [8, 26].

WRN is a member of the RecQ DNA helicase family, which includes BLM, RECQL4, RECQ1, and RECQ5 [27, 28]. RecQ helicases contribute to genome stability by regulating HR through various mechanisms [29]. BLM, in particular, has common functions with WRN, and is also phosphorylated on multiple residues by different kinases either after stress induction or during mitosis. WRN Thr-99 and −122 are phosphorylated by replicative stress induced by hydroxyurea [30]. Ser-646 is constitutively phosphorylated by Chk1, and treatment of cells with DNA damaging agents leads to decrease of the phosphorylation and relocalization of BLM to the site of DNA damage [31]. Ser-714 and Thr-766 are also phosphorylated by cdc2 in mitosis [32]. In contrast to Ser-440 and −467 in the acidic region in WRN, neither of these serines and threonines is located in the acidic region in BLM. Thus, phosphorylation of the acidic region may be unique to WRN. Because phosphorylation of WRN by DNA-PK regulates its exonuclease and helicase activities [11], WRN phosphorylation may influence the dissociation of HR intermediates.

DNA-PKcs belongs to the PI-3 kinase family with ATR, ATM and other kinases, and WRN is also a substrate for ATR and ATM kinases in response to replication blockage [33, 34], raising the possibility that Ser-440 and −467 are also phosphorylated by ATR or ATM. The major phosphorylation sites by ATR are Ser-991, Thr-1152 and Ser-1256 [10]. Ser-1141, −1058 and −1292 were previously identified as phosphorylation sites by ATM[33]. Thus, Ser-440 and −467 have not been identified as substrates so far. In addition, the minimal consensus sequence of ATR or ATM is the SQ/TQ motif, and Ser-440 and −467 are followed by D. Therefore, these two serines may not be targets of ATR and ATM. Taken together, Ser-467, the major DNA-PK phosphorylation site is unlikely to overlap with the ATR and ATM phosphorylation sites. LC-MS/MS analysis detected phosphorylation at Ser-1141, however, results from *in vitro* phosphorylation excluded the serine as a substrate for DNA-PK. ATM may have already phosphorylated the WRN protein in Sf9 cells before or during protein purification, resulting in detection of background phosphorylation. ATR and ATM promote recovery from perturbed replication by differently regulating WRN at defined moments of the response to replication fork arrest [10]. Thus, these three PI-3 kinases respond to incidents on DNA through phosphorylation of WRN at different positions and at different times.

## MATERIALS AND METHODS

### Cells

HEK293 cells, 293T cells and HeLa cells were grown in monolayer in Dulbecco's Modified Eagle Medium (DMEM) supplemented with 10% fetal bovine serum, 50 μg/ml streptomycin and 50 U/ml penicillin. SV40-transformed Werner (AG11395) cells were grown in monolayer in minimum essential medium (MEM) supplemented with 10% fetal bovine serum, 50 μg/ml streptomycin and 50 U/ml penicillin, 1% amino acids, 1% vitamins and 2 mM L-glutamine (Life Technologies, Carlsbad, CA, USA).

### Proteins

The GST-WRN fragments were overexpressed in *Escherichia coli* BL21(DE3) pLysS and purified as previously described [35, 36]. His-tagged full length WRN [37], His-tagged WRN fragment (1-368)[37], untagged, human Ku 70/86 [38] and DNA-PKcs[39] were purified using a baculovirus expression system as described previously.

### Vectors

Site-directed mutagenesis was performed on pEGFP-WRN[40] in order to change amino acid residues of WRN, Ser-440, −467, ^−−^478 and −1141, to alanine using the QuikChange II Site-Directed Mutagenesis Kit or the QuikChange Multi Site-Directed Mutagenesis Kit (Agilent Technologies, Santa Clara, CA, USA). Mutations were confirmed by sequencing.

### *In vitro* phosphorylation

7.5 μM WRN fragment and 0.2 μM Ku 70/86 in 10 μl of kinase buffer [50 mM KCl, 1 mM dithiothreitol, 25 mM Hepes-KOH (pH 7.5), 5 mM MgCl_2_, 250 μM ATP, 1 μCi of [γ-^32^P]ATP, and 100 ng of sonicated calf thymus DNA] were incubated with 0.05 μM DNA-PKcs at 30°C for 15 min. The reactions were stopped by the addition of 1% SDS. The resulting products were analyzed by 4-15% SDS-PAGE. The proteins on the gel were transferred to a PVDF membrane. The membrane was analyzed on a Typhoon Phophorimager (GE Healthcare, Piscataway, NJ, USA) and quantified using Imagequant software (GE Healthcare), followed by amido black staining.

### *In vivo* phosphorylation

Phosphorylated WRN was detected by radio-labeling as previously reported^11^ or by probing with anti-phosphoserine antibody. Briefly, 293T cells were transfected with pEGFP-WRN vector using Polyfect Transfection Reagent (Qiagen, Valencia, CA, USA), and were incubated with phosphate-free DMEM (Life Technologies) for 1 hour to exhaust the pool of endogenous phosphate. The cells were then incubated with phosphate-free medium containing 100 μCi of [^32^P] orthophosphate (GE healthcare) and 5 μg/ml bleomycin (Sigma-Aldrich, St Louis, MO, USA) for 5 hours. In some experiments, 25 μM wortmannin (Sigma-Aldrich) or 20 μM NU7026 was added during the incubation period. The cells were then lysed with RIPA buffer A [150 mM NaCl, 1% Triton, 0.1% SDS, 10 mM Tris (pH 8.0), 0.5% sodium deoxycholate, 0.2 mM phenylmethylsulfonyl fluoride, 20 μg/ml aprotinin, 10 μg/ml leupeptine, 1 mM sodium orthovanadate and 10 units/ml DNase I (New England Biolabs, Beverly, MA, USA)] supplemented with phosphatase inhibitors (1:1,000, Sigma-Aldrich). After centrifugation, the supernatant was precleared with Recombinant Protein G Agarose (Life Technologies) and incubated with 4 μg of polyclonal rabbit anti-WRN antibody (sc-5629, Santa Cruz Biotechnology, Dallas, TX, USA) for 16 hours. The immuno complexes were collected by adding Recombinant Protein G agarose and washed three times with RIPA buffer A. Immunoprecipitated proteins were eluted with SDS and analyzed by 4-15% SDS-PAGE. The proteins were transferred to a PVDF membrane and analyzed on a Typhoon Phophorimager using ImageQuant TL software (GE Healthcare). Western blot was performed using mouse monoclonal anti-WRN antibody (BD Biosciences, San Jose, CA, USA).

HEK293 cells were transfected with pEGFP-WRN vector using FuGENE 6 (Promega, Madison, WI, USA), and were incubated with 5 μg/ml bleomycin (Sigma-Aldrich) for 5 hours. The cells were then lysed with RIPA buffer B [150 mM NaCl, 0.05% NP-40 and 50 mM Tris (pH 7.5)] supplemented with protease inhibitor cocktail without EDTA (Roche, Basel, Schweiz), and phosphatase inhibitors I (Sigma-Aldrich). After centrifugation, the supernatant was incubated with 50 μl of anti-GFP magnetic beads (D153-9, MBL, Nagoya, Japan) for an hour. The complexes were washed three times with RIPA buffer B. Immunoprecipitated proteins were eluted with SDS and analyzed by 4-15% SDS-PAGE. The proteins were transferred to a PVDF membrane. Western blot was performed using mouse monoclonal anti-WRN antibody (BD Biosciences) or mouse anti-phosphoserine antibody (ab-6639, Abcam, Cambridge, MA, USA).

### Identification of phosphorylation sites

*In vitro* phosphorylated WRN were separated by 4–15% SDS–PAGE. The gel was then stained with Coomassie brilliant blue, and the band was then excised and subjected to in-gel tryptic digestion (Thermo Scientific, Waltham, MA, USA) according to the manufacturer's instructions. To identify the Ser/Thr phosphorylation site of WRN, the digests was subjected to immobilized metal affinity chromatography (IMAC) and analyzed by capillary-LC-MS/MS.

### Laser microirradiation and confocal microscopy

We employed a Nikon Eclipse TE2000-E (Nikon, Tokyo, Japan) with a Yokogawa CSU 10 Spinning Disk head (Yokogawa, Tokyo, Japan) for confocal microscopy. The set-up integrated a Stanford Research Systems NL100 nitrogen laser (Sunnyvale, CA, USA) by Micropoint ablation system (Photonic Instruments, St Charles, IL, USA). HeLa cells were grown on 35 mm glass bottom culture dishes (MatTek corporation,Ashland, MA, USA) and 24 hours before targeting, transfected with pEGFP-WRN vector using Lipofectamine 2000 (Life Technologies). The power of the laser is attenuated through Improvison's Volocity software 5.1 (PerkinElmer, Waltham, MA, USA) in terms of percent intensity. Positions internal to the nuclei of cells were targeted via a 40× oil objective lens. 14% laser intensities were used to produce DSBs. Images were captured and analyzed using Volocity software (PerkinElmer).

### Detection of WRN foci after etoposide exposure

AG11395 cells were transfected with pEGFP-WRN vector using FuGENE HD (Promega). After 48 hours, cells were incubated with 35 μM of etoposide (Sigma-Aldrich) for 3 hours and allowed to recover for 24 hours, followed by fixation with 4% paraformaldehyde. EGFP-WRN foci were detected using a Leica DMLB 100S microscope under 100× magnification.

### Comet assay

The alkaline single-cell gel electro-phoresis assay (comet assay) was performed to detect DSBs as previously reported with minor modifications [41, 42]. Briefly, cells transfected with WRN expressing vector were treated with etoposide for 3 h. Cells were mounted in agarose gel on slides. The slides were incubated in lysis solution [2.5 M NaCl, 100 mM EDTA, 10 mM Tris (pH 10), 10% DMSO and 1% Triton X-100] for 1 h at 4 °C. After washing with 0.4 M Tris (pH 7.5) for 20 min, the slides were electrophoresed in buffer [0.3 M NaOH and 1 mM EDTA] at 25 V (0.78 V/cm) for 20 min, and were stained with ethidium bromide (20 μg/ml). Nuclei were randomly chosen and were analyzed under fluorescence microscope (Leica Microsystems, Wetzlar, Germany). The percentages of tail DNA were evaluated using Komet Assay software (Komet version 5.5, Kinetic Imaging Ltd., Nottingham, United Kingdom) under 400× magnification.

### Cell proliferation assay

AG11395 cells were seeded at a density of 2 × 10^5^ cells per well in 24-well plates and incubated overnight. Cells were transfected with pEGFP-WRN vector using FuGENE HD. After 24 hours of transfection, cells were exposed to 0, 15, 25 or 35 μM etoposide for 3 hours and incubated for another 24 hours in fresh medium. The cell proliferation was evaluated with standard (3, 4, 5-dimethylthiazol-2-yl)-2, 5-diphenyltetrazolium bromide (MTT) assay[43].

### Statistical analysis

All statistical analyses were performed using Microsoft excel analysis tools. The data analysis was conducted using paired *t* test. The statistically significant level was reached at p< 0.05.

## SUPPLEMENTARY FIGURES


