# Supplementary material for: Serines 440 and 467 in the Werner syndrome protein are phosphorylated by DNA-PK and affects its dynamics in response to DNA double strand breaks
Source: Aging (Albany NY). 2014 Jan 14;6(1):70–81. doi: 10.18632/aging.100629 (PMC3927811; doi:10.18632/aging.100629)
Supplement: Supplementary file 1 [file aging-06-070-s001.pdf]

SUPPLEMENTARY FIGURES

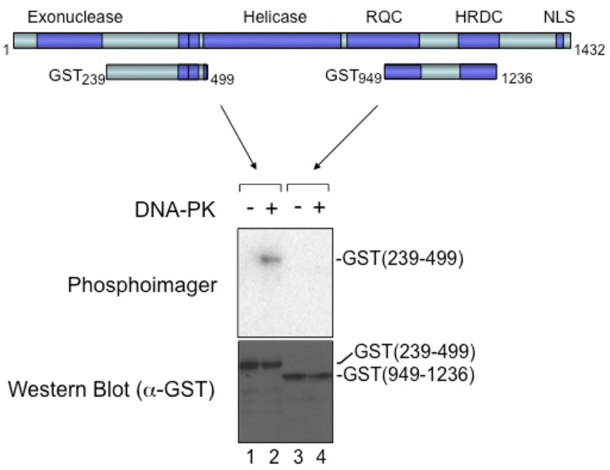

Supplementary Figure 1. WRN (949-1236) is not phosphorylated by NA-PK. Purified GST-tagged WRN fragment (239-499) and (949-1236) are used as ubstrates for *in vitro* phosphorylation assays. The phosphorylation was visualized (upper panel) and GST-WRN fragments are blotted with anti-GST antibody (lower panel).

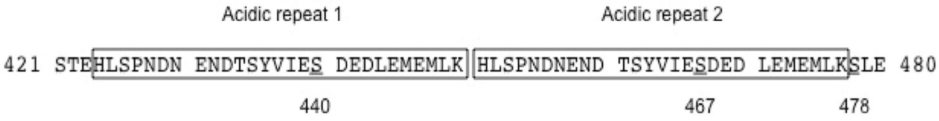

Supplementary Figure 2. Potential WRN phosphorylation sites around acidic repeats. Ser-440 and -467 locates in acidic repeats and Ser-478 locates right after the repeats.

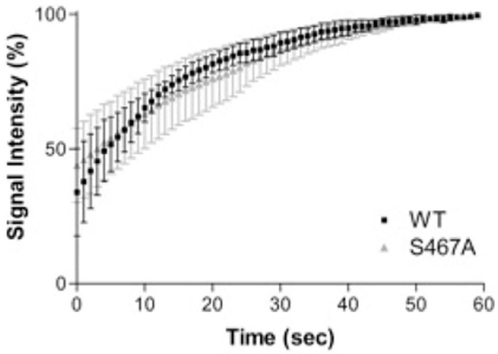

Supplementary Figure 3. Recruitment kinetics of GFP-WRN wild type and S467A to laser-induced DSBs. HeLa cells overexpressing either EGFP-WRN wild type or S467A were laser-irradiated to produce DSBs. Signal intensity of EGFP-WRN wild type (square) and S467A (triangle) at irradiated sites were plotted.
